# CD4‐Derived Double‐Negative T Cells Ameliorate Alzheimer's Disease‐Like Phenotypes in the 5×FAD Mouse Model

**DOI:** 10.1111/cns.70187

**Published:** 2025-01-23

**Authors:** Yuanzi Xie, Jing Liu, Zongren Hou, Huan Wang, Kailun Liu, Xiaowei Chen, Zhen Fan, Da Li, Can Li, Yuhualei Pan, Yushang Zhao, Yanbing Zhu, Baoyang Hu

**Affiliations:** ^1^ State Key Laboratory of Stem Cell and Reproductive Biology, Institute of Zoology Chinese Academy of Sciences Beijing China; ^2^ University of Chinese Academy of Sciences Beijing China; ^3^ Savaid Medical School University of Chinese Academy of Sciences Beijing China; ^4^ Beijing Clinical Research Institute Beijing Friendship Hospital, Capital Medical University Beijing China; ^5^ Department of Neurology Beijing Friendship Hospital, Capital Medical University Beijing China; ^6^ Institute of Biophysics Chinese Academy of Sciences Beijing China; ^7^ Institute for Stem Cell and Regeneration Chinese Academy of Sciences Beijing China; ^8^ Beijing Institute for Stem Cell and Regenerative Medicine Beijing China

**Keywords:** Alzheimer's disease, Aβ plaques, CD4+ T cell‐derived double‐negative T cells, cell therapy, TCRαβ^+^NK1.1^−^CD4^−^CD8^−^

## Abstract

**Background:**

Alzheimer's disease (AD) is a debilitating neurodegenerative disorder that is difficult to predict and is typically diagnosed only after symptoms manifest. Recently, CD4^+^ T cell‐derived double‐negative T (DNT) cells have shown strong immuno‐regulatory properties in both in vitro and in vivo neuronal inflammation studies. However, the effectiveness of DNT cells in treating on AD are not yet fully understood.

**Objective:**

This study's aims were three‐fold, to (1) evaluate the efficacy of CD4^+^ T cell‐derived DNT cells treatment on AD mice, (2) understand how DNT treatment make changes in different cell types of 5FAD mice, (3) identify the side effects of DNT treatment.

**Methods:**

We performed tail vein injection of transformed and amplified CD4^+^ T cell‐derived DNT cells into 5 × FAD mice, while using WT mice and saline injection 5FAD mice as controls. DNT suspensions or NaCl alone were administered to 5 × FAD mice at the 6 months of age. For intravenous injection (*n* = 10 for both DNT and control injections), 5 × FAD mice were injected with a total of 5 × 10^6^ DNT cells suspended in 200 μL of 0.9% NaCl or 0.9% NaCl alone via the lateral tail vein. Behavioral tests and pathology tests were carried out 30 days after cell transplantation.

**Results:**

Through qualitative analysis, we identified 6 main themes. DNT from young wild‐type mice enhance the capability of spatial learning and memory in AD mice. DNT cell treatment rejuvenates the microglial function. DNT cell treatment improves the state of oligodendrocytes. DNT cell treatment finetunes the activation of the immune system. DNT cell treatment improves the synaptic plasticity and increases the complexity of neurons. DNT cell treatment reduces the density of amyloid Beta plaques deposition in the cortex and hippocampus of 5 × FAD mice.

**Discussion:**

The findings from this study reveal that DNT treatment improved spatial memory and learning abilities, reduced Aβ deposition, and enhanced synaptic plasticity, contrasting with previous reports on thymus‐derived DNT cells. Additionally, CD4^+^ T cell‐derived DNT therapy exhibited anti‐inflammatory effects and modulated microglial function, promoting a neuroprotective environment. Notably, DNT treatment also reduced tau pathology by decreasing levels of abnormally phosphorylated tau. These findings suggest that CD4^+^ T cell‐derived DNT cells hold therapeutic potential for AD, effectively targeting both Aβ and tau pathologies.

## Graphical abstract

We focused on a subset of CD4‐derived double‐negative T (DNT) cells called TCRαβ^+^NK1.1^−^CD4^−^CD8^−^ for treating Alzheimer's disease, and we found their injection can improve cognition of the 5×FAD mouse model. We thus identify DNT cell transplantation as a potential therapeutic approach for future AD treatments.

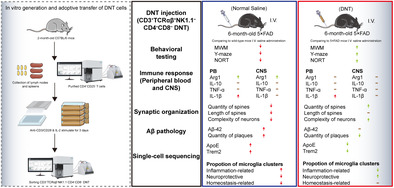

## 1 Introduction

Alzheimer's disease (AD) is a debilitating neurodegenerative disorder that is difficult to predict and is typically diagnosed only after symptoms manifest, placing a significant burden on patients, families, and society [1]. The disease is characterized by accumulating pathological Aβ plaques, which disrupt neurological communication and impair cognitive function [2]. In addition, systemic inflammation and pathological changes to microglia in the brain have been observed in AD patients [3]. While current drugs can only slow the pathological process to some extent [4], novel therapeutic methods and targets are needed for the treatment of AD.

Cell therapy using autologous or allogeneic cells has shown promise in repairing damaged tissues and organs, and is widely used to treat conditions such as bone marrow failure, cirrhosis, femoral head necrosis, malignancy, and myocardial infarction. Infusion of mesenchymal stem cells (MSCs) or neural stem cells (NSCs) has shown positive outcomes in treating AD [5]. However, the widespread application of these cells for therapy is limited by their source of derivation.

Double‐negative T cells are a unique subset of T lymphocytes characterized by the lack of CD4 and CD8 surface markers, distinguishing them from conventional CD4^+^ helper T cells and CD8^+^ cytotoxic T cells. DNT cells are typically identified by their TCRαβ^+^CD3^+^CD4^−^CD8^−^ phenotype in mice and TCRαβ^+^CD3^+^CD4^−^CD8^−^CD56^−^ in humans. Despite representing only 1%–3% of peripheral T cells in both mice and humans, DNT cells have attracted significant attention due to their diverse roles in immune regulation, inflammation, and tissue homeostasis [6]. Based on their origin, DNT cells can be classified into the following types: (1) Thymus‐derived DNT cells: These cells develop directly in the thymus and represent an intermediate population during T‐cell maturation as CD4^−^CD8^−^ double‐negative stages. They are primarily involved in maintaining immune tolerance and regulating immune responses, with some escaping thymic selection and migrating into peripheral blood. (2) CD4^+^ or CD8^+^T Cell‐Derived DNT Cells: Under specific conditions, CD4^+^ or CD8^+^T cells can lose their CD4 or CD8 expression and differentiate into DNT cells [7]. CD4‐derived DNT cells have shown strong immunoregulatory properties in both in vitro and in vivo studies, demonstrating significant potential in the treatment of autoimmune diseases, such as type 1 diabetes [8], and in promoting transplant tolerance and regulating inflammation‐related diseases [9].

DNT cells have attracted attention in Alzheimer's disease (AD) research due to their unique immunoregulatory characteristics and potential roles in neuroinflammation and neurodegeneration. Previous studies have shown an increase in the percentage of CD3^+^CD4^−^CD8^−^ (DNT) cells in the blood of 3xTg‐AD mice [10]. The injection of thymus‐derived DNT cells into the APP/PS1‐AD mouse model was found to exacerbate cognitive impairment [11]. However, the effects of CD4^+^ T cell‐derived DNT cells in AD remain unknown.

In this study, we injected transformed and amplified CD4^+^ T cell‐derived DNT cells into 5×FAD mice via tail vein injection and examined their pathological phenotype. We found that DNT treatment improved spatial memory and learning ability reduced the pathological phenotype of Aβ deposition, and significantly improved synaptic plasticity in mice, in addition to having some inflammatory protective function. Ultimately, we found that CD4^+^ T cell‐derived DNT treatment may shift microglia status towards neural relevance in a subset of AD mice. In further analysis, we observed that DNT treatment also had an impact on tau pathology. Specifically, DNT therapy reduced the levels of abnormally phosphorylated tau in the tau‐293T cell line. These findings suggest that CD4+ T cell‐derived DNT treatment may be a potential therapeutic option for Alzheimer's disease, particularly in addressing both Aβ and tau pathologies.

## 2 Materials and Methods

### 2.1 Animals

The 5×FAD mice (MMRRC ID 034848‐JAX‐008730) in C57BL/6 background were obtained from the Jackson Laboratory. These mice overexpress mutant human Aβ (A4) precursor protein (APP) and human presenilin‐1 (APPSwFlLon, PSEN1*M146L*L286V). Mice were housed at the Laboratory Animal Center of the Institute of Zoology under standard conditions, including a 12:12‐h light/dark cycle, and were allowed free access to food and water. All animal experiments were approved by the Animal Care and Use Committees of the Institute of Zoology, Chinese Academy of Sciences.

### 2.2 In Vitro Generation and Adoptive Transfer of DNT Cells

The DNT cells were converted and amplified in vitro as in the previous studies [12, 13, 14]. CD3^+^TCRβ^+^CD4^−^CD8^−^NK1.1^−^ DNT cells were sorted, and DNT cells (5 × 10^6^) were adoptively transferred intravenously into 5×FAD mice after 30 min or 2 h.

### 2.3 Transplantation of DNT Into 5×FAD

DNT suspensions or NaCl alone were administered to 5×FAD mice at the age of 6 months. For intravenous injection (*n* = 10 for both DNT and control injections), 5×FAD mice were injected with a total of 5 × 10^6^ DNT suspended in 200 μL of 0.9% NaCl or 0.9% NaCl alone through the lateral tail vein. Behavioral tests and pathology tests were carried out 30 days after cell transplantation.

### 2.4 Morris Water Maze

The water maze was a circular pool (120 cm in diameter, 60 cm in height) with a white inner surface. The escape platform (10 × 10 cm) was fixed in the center of one quadrant and submerged 1 cm below the water surface. In training sessions, mice could navigate the tank to find the hidden platform. If a mouse failed to find the platform within 60 s, it was gently guided to the platform and allowed to stay there for 25 s. Each mouse performed eight training trials per day, starting from different quadrants, for 5 days. Test sessions were performed 24 h after the last training trial. The test session was a single probe test in which the platform was removed and mice were allowed to swim in the tank for the 60 s. Behaviors were analyzed by video tracking software (EthoVision, Noldus, Netherlands). Latency to find the platform during the training trials and the time spent in each quadrant during the test session were recorded.

### 2.5 Y Maze Test

The Y‐maze apparatus consisted of three radial 30‐cm‐long arms (named starting, novel, and other arms) originating from the central space to form a “Y” shape. Mice were placed into the starting arm to explore the maze based on the rodent's innate curiosity to explore unexplored areas. Briefly, mice were placed into the starting arm to explore and allowed 5 min to freely locate the novel arms using spatial clues (training period). After a 2‐h interval, mice were placed into the Y‐maze again as part of the training period protocol to evaluate spatial memory. Time, distance, entry times, and movement tracks were recorded by an automated video tracking system.

### 2.6 Novel Object Recognition

Novel object recognition is widely used in rodents to measure short‐term memory and learning, the preference for novelty, and the hippocampus's influence on the recognition process [15]. The test was performed in a square‐shaped open‐field box with objects located opposite the starting point. Briefly, mice were allowed to explore two identical objects (cylinders) in the open field for 10 min (learning period). After a 3‐h interval, mice were allowed to explore one familiar object (cylinder) and one novel object (cuboid) as part of the learning period protocol. The time spent exploring familiar and novel objects and the movement tracks of the mice were recorded using a tracking system.

### 2.7 Open‐Field Test

The open‐field test measures the exploration of a new environment and anxious behavior and is based on the idea that mice naturally prefer to be near a protective wall rather than exposed to danger out in an open field. The test was performed in a square‐shaped open‐field box (50 × 50 cm) comprising an inside square (45 × 45 cm) as the “center area” and an outside square as the “surrounding area.” Each mouse was gently placed on the floor and allowed to freely explore the area for 10 min to investigate their spontaneous locomotor activity. Their overall time spent, distance traveled and movement tracks in the center and surrounding areas were measured by a tracking system.

### 2.8 Western Blotting

Protein samples were separated by 4%–20% SDS‐PAGE, blotted onto a polyvinylidene fluoride membrane (Millipore), blocked for 60 min in 5% milk, and incubated overnight at 4°C with mouse monoclonal anti‐TUJ1 (1:1000; 801,201; BioLegend), mouse monoclonal anti‐β‐Actin (1:4000; A5441; Sigma), mouse monoclonal anti‐GAPDH (1:4000; AF0006; Beyotime) antibodies, rabbit recombinant monoclonal anti‐synaptophysin (1:1000; ab32127; Abcam) antibodies, rat recombinant monoclonal anti‐CD68 (1:1000; ab53444; Abcam) antibodies, mouse purified anti‐Aβ1‐16 (6E10) (1:500; 803,004; BioLegend), rabbit recombinant monoclonal anti‐APOE (1:1000; ab183597; Abcam) antibodies, and mouse purified anti‐Aβ42 (1:500; 899910; BioLegend). After three washes with TBST, the membranes were incubated with horseradish peroxidase‐conjugated goat anti‐mouse or goat anti‐rabbit secondary antibodies at room temperature for 2 h. The immunoreactive bands were detected using an enhanced chemiluminescence reagent (ECL, Pierce) and quantified using ImageJ software.

### 2.9 Immunofluorescence

Mice were anesthetized with 2.5% avertin (200 mg/kg body weight) and then perfused with cold PBS followed by 4% paraformaldehyde (PFA). The brains were subsequently removed and post‐fixed in 4% PFA overnight and then dehydrated with 30% sucrose. Finally, the brains were coronally sectioned into 35‐μm‐thick slices using a cryostat (Leica SM2010R) and stored at −20°C in cryoprotective storage solution (125 mL) ethylene glycol, 125 mL of glycerol, and 150 mL of 0.1 mol/L phosphate buffer until use. For immune histochemical staining, the sections were washed three times with PBS, blocked using 5% BSA and 1% Triton X‐100 in PBS for 2 h at room temperature, and incubated with primary antibodies (in 1% BSA and 0.2% Triton X‐100 in PBS) overnight at 4°C. The primary antibodies used include rabbit polyclonal anti‐Iba1 (1:1000; 01919741; Wako, Saitama, Japan), rat recombinant monoclonal anti‐Myelin Basic Protein (1:200; ab7349; Abcam) antibodies, rat recombinant monoclonal anti‐CD68(1:50; ab53444; Abcam) antibodies, mouse purified anti‐Aβ1‐16 (6E10) (1:500; 803004; BioLegend), and anti‐doublecortin (1:100; ab18723; Abcam). After washing, the sections were incubated with secondary antibodies conjugated to Alexa Fluor 594 in a blocking solution. The images were captured with the confocal microscope (Leica).

### 2.10 Thioflavin‐S Staining

For the detection of Aβ plaques, brain sections were first incubated with 0.1% thioflavin‐S (Thioflavin‐S, Sigma) in the dark for 5 min in 50% ethanol, followed by two washes with 50% ethanol and three washes with PBS, and then subjected to antibody staining as described above.

### 2.11 Golgi Staining

Mice were sacrificed to get the whole brain tissues and cut off the olfactory bulbs and cerebellum. Then the tissues were administrated following the steps of the Golgi staining kit (FD Rapid GolgiStain Kit, PK401, FD NeuroTechnologies). The sections were shot and analyzed by software (Leica Aperio VESA8, Leica, Germany).

### 2.12 RNA‐Seq Analysis

RNA and library preparation, clustering, sequencing, and data analyses were performed by the BGI Experimental Department. Sequencing libraries were generated using the NEB Next Ultra RNA Library Prep Kit for Illumina (NEB) according to the manufacturer's protocol, and index codes were added to attribute sequences to each sample. After cluster generation, the prepared libraries were sequenced on an Illumina platform, and 125 bp/150 bp paired‐end reads were generated. A differential expression analysis between the two groups was performed using the DESeq2 package in R (1.16.1). Genes with an adjusted *p*‐value of < 0.05 (obtained by DESeq2) were considered to be differentially expressed. A corrected *p*‐value of 0.05 and an absolute fold change in 2 were set as the thresholds for significantly differential expression. The KEGG is a database resource for understanding a biological system's high‐level functions and utilities. Data were analyzed using the cluster Profiler package in R to test the enrichment of DEGs in KEGG pathways. A gene ontology (GO) enrichment analysis was implemented with the same package.

### 2.13 Isolation of Single Cells From Brain Tissues

The brain tissues were harvested using fine forceps and digested [0.1 mg/L collagenase IV, 1% calf serum in phosphate‐buffered saline (PBS)] at 37°C for 40 min. Single‐cell suspensions were filtered using a 70 μm cell strainer and washed with MACS buffer (5 g/L bovine serum albumin, 2 mM EDTA, PBS), which was used for subsequent experiments.

### 2.14 Data Analysis

All statistical analyses in this study were performed using Graphpad Prism 9.1.0 (Graphpad Software Inc.). Paired *t*‐test or unpaired Student's *t*‐test was used to compare two groups with normally distributed data. One‐way ANOVA was used for comparing data among multiple groups, followed by Tukey's post hoc multiple‐comparisons test. For data that did not follow a normal distribution, non‐parametric tests were employed. Measurement data were presented as mean ± standard error of mean, and *p* < 0.05 was considered statistically significant.

## 3 Results

### 3.1 DNT From Young Wild‐Type Mice Improves the Capability of Spatial Learning and Memory in AD Mice

**FIGURE 1:**
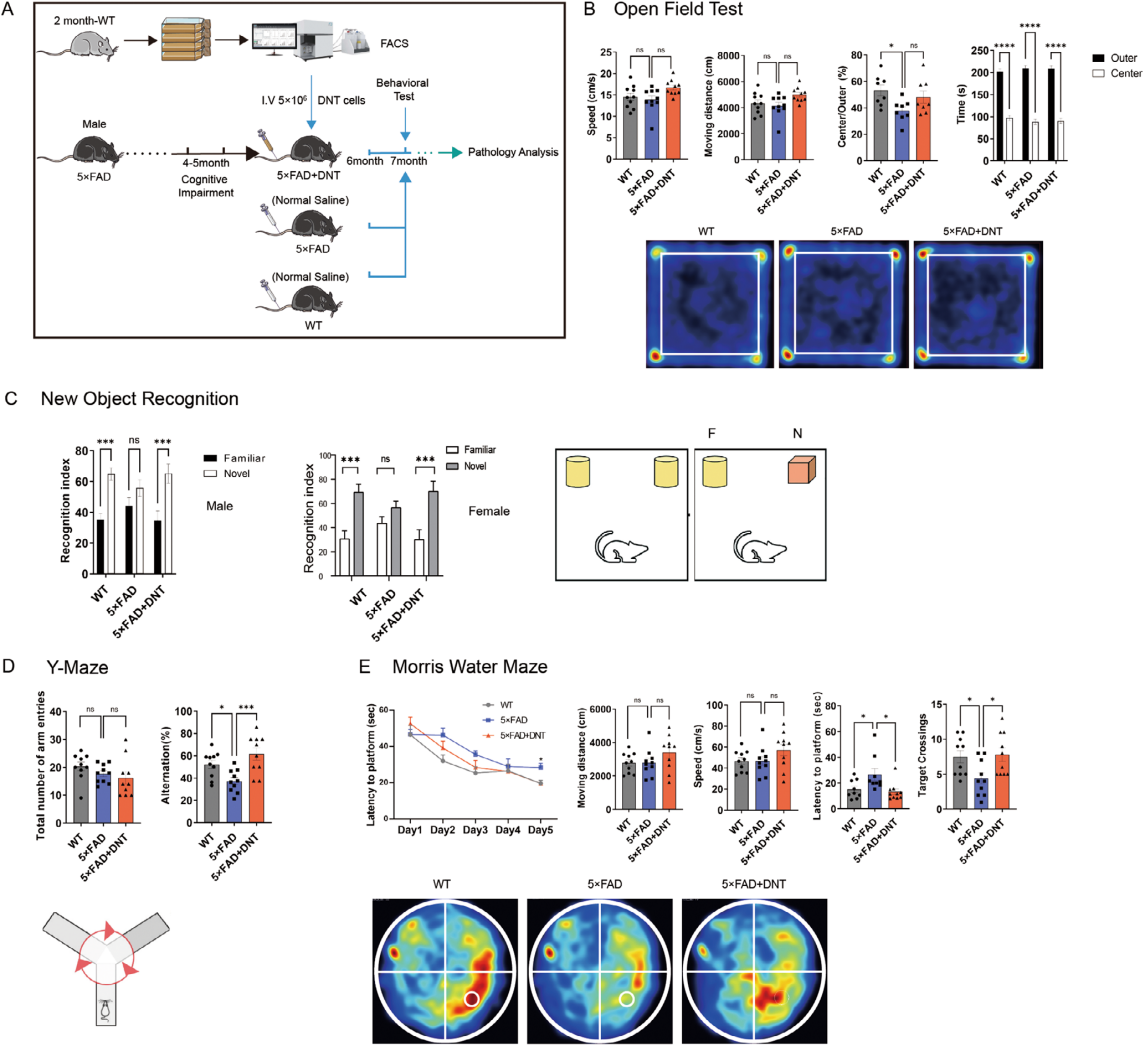
(A) Experiment design for DNT treatment (WT, wild‐type mice intravenous (IV) saline administration; 5×FAD, 5×FAD mice IV saline administration; 5×FAD+DNT, 5×FAD mice IV DNT administration) and behavior assessment in Alzheimer's disease. (B) Results of the open‐field test for the three groups indicated that there were no differences in moving speed and distance among these groups. (C) Schematic of the new object recognition test (NORT) (left, male and female), recognition index [percent time exploring familiar novel objects in the NORT (right). (D) Schematic of the Y‐maze (lower), the number of arm entries (left), and the percentage of alterations [(actual alterations/maximum alterations‐2) × 100%] (right). (E) Results of the Morris Water Maze for these three groups. The mean of daily escape latencies (upper left) and latency to the platform of test and target crossing times (upper right), the integrated route heat map (lower) were shown. There was no difference in swimming distance and speed (upper 2 and 3). In each graph, *n* = 9–10 mice per group; all data are shown as means ± SEM. All comparisons were made by one‐way ANOVA with Tukey's multiple comparison testers, ns, nonsignificant, *p < 0.05; **p < 0.01; ***p < 0.001; ****p < 0.0001. DNT, double‐negative T cells.

To investigate whether DNT from young wild‐type mice could improve cognition in AD mice, we isolated DNT from the lymph nodes and spleens of 2‐month‐old wild‐type mice, expanded the cells in vitro, and infused them into 6‐month‐old 5×FAD mice through tail vein injection (5 × 10^6^ cells/mouse). We injected equal volumes of saline into either WT or 5×FAD mice as controls. After a month of injection, we conducted behavior tests to evaluate cognitive function (Figure 1A).

In the open field test, all groups of male mice exhibited no significant difference in moving distance and speed, indicating similar levels of spontaneous activity behavior. The mice spent similar amounts of time in open space and at the edge, suggesting comparable exploration competence and no anxiety trend between the AD mice and the DNT‐treated mice (Figure 1B). However, we found that female mice were more active than the WT group and DNT‐treated group (Figure S1A), the results consistent with previous research [16].

The DNT‐treated group also exhibited a markedly increased novel object exploration and showed better spontaneous alternation in the Y‐maze test in the new object recognition test (NORT) than the control AD mice no matter male or female mice, indicating that DNT injection improved short‐term memory and cognition (Figure 1C,D).

In the Morris water maze (MWM) test to evaluate spatial orientation learning and memory ability, we randomly trained the three groups of mice in four directions a day for 5 days. While all three groups of mice exhibited indistinct distance and speed, the DNT‐treated group showed a significantly shorter latency to the platform and more target crossings than the control AD mice, indicating that DNT treatment improved spatial orientation learning and memory ability in AD mice (Figure 1E).

Overall, the DNT‐treated AD mice displayed improved spatial learning and memory ability compared to the 5×FAD group, indicating the remarkable therapeutic effect of DNT from young wild‐type mice for AD treatment.

### 3.2 DNT Cell Treatment Rejuvenates the Microglial Function

**FIGURE 2:**
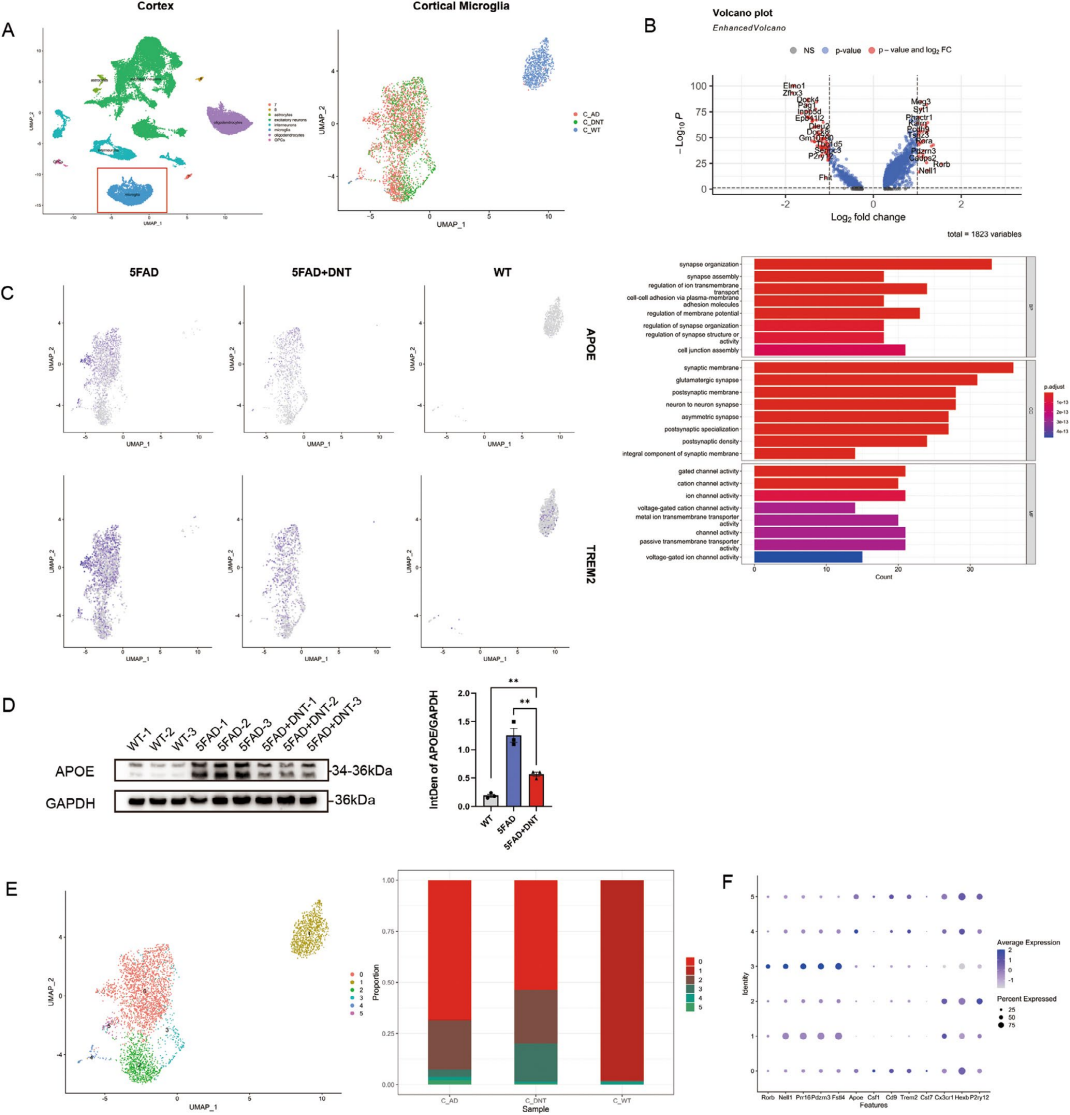
(A) Unbiased identification of cell‐type heterogeneity in the DNT‐treated mice cortex (left), and microglial cell populations are marked in red. UMAP harmony shows the microglia cluster of the three groups of mice (right). *n* = 3 mice per group for single‐cell sequencing, analyzed by Seurat (R package, 4.1.0). (B) Volcano plot depicting changes in gene expression in microglia owing to DNT treatment compared to 5×FAD mice. The *x*‐axis corresponds to log_2_ (fold change in gene expression), and the *y*‐axis indicates the adjusted *p*‐value. The genes colored red have the symbol as follows: Log_2_ fold change > 1 or < −1 and adjusted *p*‐value < 0.05, and genes colored blue meet the standard of the log_2_ fold change < 1 or > −1 and adjusted *p*‐value < 0.05. Bar graph showing enriched ontology terms for significantly upregulated genes (log_2_ fold change > 1 and *p*‐value < 0.05) in DNT‐treated mice relative to 5×FAD mice microglia (lower). (C) UMAP plots of the microglia in (A, right). UMAP plots are colored by the expression of key marker genes, *Apoe* and *Trem2*. (D) Western Blotting of APOE expression in the whole brain tissue of three groups, every lane represents one mouse, and the statistical graph (right) is shown according to the intensity. *n* = 3 mice per group; mean ± SEM, ns, nonsignificant, **p < 0.01; unpaired Student's *t*‐test. (E) UMAP plots of the microglia in (A, right). Distribution of mice cortical microglia into six distinct clusters (left). Quantification of the different clusters of microglia (right), the color bar represents the average expression of genes, and the circle size represents the percentage of gene expression. The numbers 0–5 are consistent in the three graphs, representing the six different microglia clusters. (F) Markers labeled the six clusters are shown. The numbers of cell clusters labeled in the (E, F) correspond.

In neurodegenerative diseases, microglia lose their ability to prune synapses and phagocytose pathological plaques [17]. However, DNT cell treatment improves cognition and memory in mice. To investigate whether microglia function was restored after DNT cell treatment, we conducted single‐cell sequencing of the cortex and hippocampus of the mice in each group. We found significant differences in microglial fractionation among the three groups of mice (Figure 2A). Among the differentially expressed genes in microglia between the DNT cell‐treated group and the 5xFAD group, we observed a significant change in *Elom1* (Figure 2B), a gene that regulates synaptic pruning [18, 19]. Additionally, we observed the downregulation of some inflammation‐ and apoptosis‐related factors and *P2ry12* (Figure 2B), a gene associated with microglial homeostasis, although the impact of this gene on microglia is currently unclear. GO analysis of the upregulated genes revealed a significant increase in synaptic remodeling (Figure 2B). Moreover, we observed altered expression of *Apoe* and *Trem2*, genes representative of AD pathology (Figure 2C), and the expression in the whole brain of APOE is significantly decreased in the DNT‐treated group (Figure 2D). *Apoe* is an apolipoprotein, and its dysregulation in microglia in AD is driven by the *Apoe4* allele, which is a risk gene for AD. DNT cell treatment significantly decreased the expression of *Apoe* in microglia. We also observed a decrease in *Trem2* expression in microglia of DNT‐treated mice, suggesting that injection of DNT cells reduced the risk of exacerbation of AD [20, 21].

DNT cell treatment of AD mice caused significant neuro‐synaptic remodeling, and we classified the three microglia groups into six clusters based on their representative characteristics (Figure 2E). Specific representative genes related to neuronal growth and differentiation, such as *Rorb*, *Nell1*, *Prr16*, *Pdzrn3*, and *Fstl4* (Figure 2F), were expressed in some of these clusters [22, 23, 24, 25, 26, 27]. The proportion of these six microglia clusters in the mouse brain was variable, with cluster 3 significantly increased and the DAM‐related [28] cluster 0 decreased in the DNT‐treated group. Also, we sub‐populated microglia in the hippocampus and found a similar trend to microglia in the cortex (Figure S1B–E).

### 3.3 DNT Cell Treatment Improves the State of Oligodendrocytes

**FIGURE 3:**
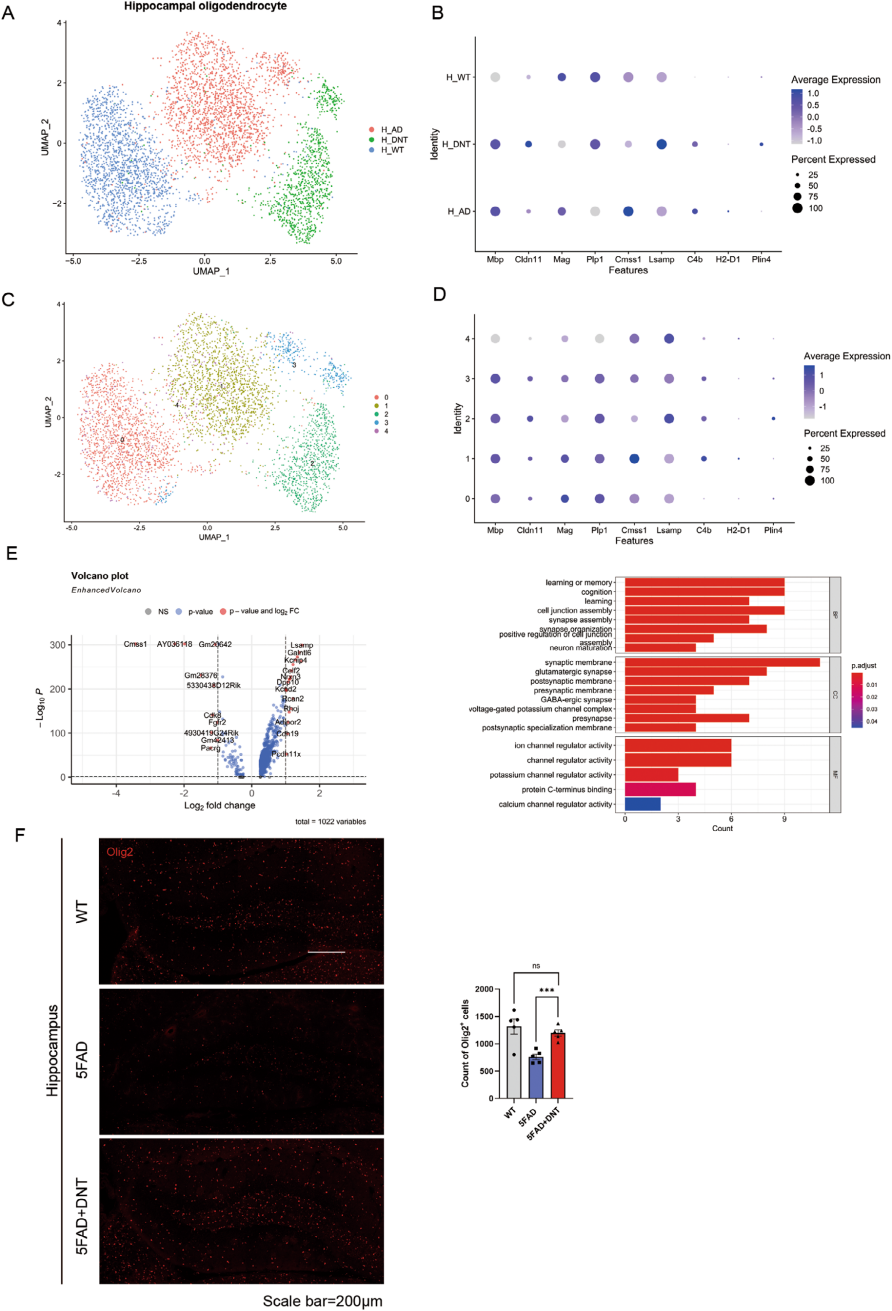
(A) UMAP harmony showing the oligodendrocyte cluster of the three groups of mice (WT, wild‐type mice intravenous (IV) saline administration; AD, 5×FAD mice IV saline administration; DNT, 5×FAD mice IV DNT administration). (B) Markers labeled the three groups of mice hippocampus oligodendrocytes are shown, the color bar represents the average expression of genes, and the circle size represents the percentage of gene expression. *n* = 3 mice per group for single‐cell sequencing, analyzed by Seurat (R package, 4.1.0). (C) UMAP plots of the oligodendrocyte in (A). Distribution of the three groups of mice hippocampal oligodendrocytes into five distinct clusters. (D) Markers label the five clusters are shown, the color bar represents the average expression of genes, and the circle size represents the percentage of gene expression. The numbers 0–4 are consistent in the two graphs, representing the five different oligodendrocyte clusters. (E) Volcano plot depicting changes in gene expression in oligodendrocytes owing to DNT treatment compared to 5×FAD mice. The *x*‐axis corresponds to log_2_ (fold change in gene expression), and the *y*‐axis indicates the adjusted *p*‐value. The genes colored red have the symbol as follows: Log_2_ fold change > 1 or < −1 and adjusted *p*‐value < 0.05, and genes colored blue meet the standard of the log_2_ fold change < 1 or > −1 and adjusted *p*‐value < 0.05. Bar graph (right) showing enriched ontology terms for significantly upregulated genes (log_2_ fold change > 1 and *p*‐value < 0.05) in DNT‐treated mice relative to 5×FAD mice oligodendrocyte. (F) Representative confocal images of *Olig2* immunostaining in the hippocampus of the three groups of mice (left) and the number of Olig2^+^ cells in the certain area were calculated (right). Scale bar = 200 μm; *n* = 5 views from 3 mice per group; mean ± SEM, ns, nonsignificant, ***p < 0.001; unpaired Student's *t*‐test.

Oligodendrocytes are myelinated cells of the central nervous system [29] and have an important role in myelin formation and repair of nerves, and it has been suggested that the subtype of oligodendrocytes in mouse models of AD is specifically altered with the pathological process [30] and is closely related to the pathological process of AD [31]. To investigate whether the recovery of cognitive ability in mice is correlated with changes in oligodendrocytes, we performed the single‐cell analysis of oligodendrocyte status in three groups of mice and found that significant improvement in oligodendrocyte status also occurred after DNT cell treatment. We found significant gene‐related differences in oligodendrocytes in the three groups of mice (Figure 3A,B), and the expression of the gene's characteristic of oligodendrocytes, such as *Plp1* and *Lsamp*, which are related to myelin composition and axon direction, was significantly higher in the DNT‐treated mice and more closely resembled the characteristics of wild‐type oligodendrocytes. Then we divided the oligodendrocytes of the three groups of mice into five clusters according to their characteristic genes (Figure 3C,D) and found the DNT‐treated mice group plays a significant part in oligodendrocytes highly expressing Plin4, a lipid droplets related gene. By comparing oligodendrocyte differential genes between DNT‐treated mice and AD mice (Figure 3E), we found that *Pacrg*, a gene related to Lewy body formation [32], was significantly downregulated, while *Kcnip4*, *Celf2*, *Nrxn3*, *Kcnd2*, and *Pcdh11x* were significantly upregulated. Both *Kcnip4* and *Kcnd2* are genes that regulate neuron excitability by regulating ion channels [33], *Celf2* regulates the *PGE2* pathway associated with *Cox2* by stabilizing its mRNA expression [34], and *Nrxn3* and *Pcdh11x* are genes associated with cell adhesion in the nervous system and play an important role in the development of the central nervous system [35, 36]. Besides, our enrichment analysis of the upregulated genes revealed that the upregulated genes were mainly involved in the regulation of ion channels (Figure 3E), which could better explain the improvement of cognition and memory in mice. To verify the change of oligodendrocyte in the hippocampus, we found the rescue of Olig2 signal in the DNT‐treated group (Figure 3F). In general, DNT treatment restored the cell counts of oligodendrocytes, which may be beneficial for neuroprotection.

### 3.4 DNT Cell Treatment Finetunes the Activation of the Immune System

**FIGURE 4:**
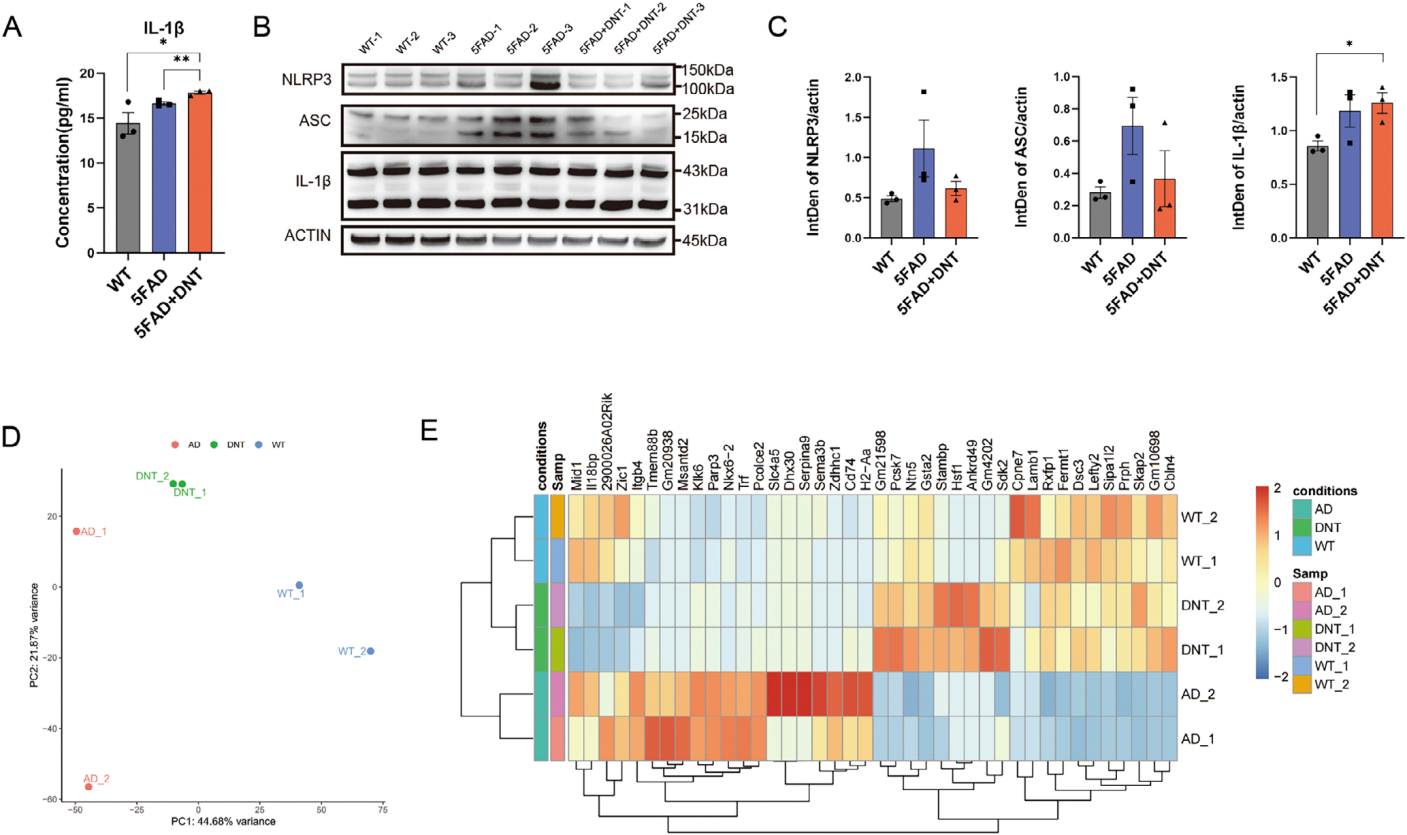
(A) Relative protein expression level of the proinflammatory‐related gene *Il‐1β* in the peripheral blood in the three groups (WT, wild‐type mice intravenous (IV) saline administration; 5FAD, 5×FAD mice IV saline administration; 5FAD+DNT, 5×FAD+DNT, 5×FAD mice IV DNT administration) were detected by ELISA. *n* = 3 mice per group; means ± SEM, ns, nonsignificant; *p < 0.05; **p < 0.01; unpaired Student's *t*‐test. (B, C) Western Blotting of anti‐inflammatory and proinflammatory cytokines in the whole brain tissue after IV DNT treatment. *n* = 3 mice per group; mean ± SEM, ns, nonsignificant, *p < 0.05; unpaired Student's *t*‐test. (D) Principal component analysis (PCA) of the saline‐treated wild‐type mice (WT1,2), and the AD mice brain tissue bulk transcriptomes with (DNT1,2) or without DNT treatment but saline treatment (AD1,2). (E) Heatmap of changed gene expression in brain tissues of the three groups of mice by RNA‐seq. Each column represents expression levels from one individual mouse, *n* = 2 mice per group. Color code presents a linear scale.

Previous studies have suggested that DNT enhances cerebral immune and inflammatory responses through the TNF‐α signaling pathway in neurodegeneration [11, 37]. However, our study found that injecting DNT cells from young wild‐type mice reversed behavioral deficits rather than exacerbating AD in mice. We, therefore, investigated the levels of inflammatory factors after DNT cell treatment. Arginase1, a representative factor of M2 macrophages, which facilitates the enzymatic production of NO and has a neuroprotective effect, has been previously identified as playing a key role in DNT cell treatment for nonalcoholic fatty liver. We performed ELISA on arginase1 and IL‐10, a major protective factor in nonalcoholic fatty liver [38], and found that neither had a protective effect on peripheral blood but a higher IL‐1β expression (Figure 4A).

Other studies have suggested that DNT cell treatment leads to elevated TNFα expression, stimulates NLRP3 inflammasome activation, and may increase IL‐1β expression [11, 39]. To determine whether DNT cell treatment caused more severe inflammation, we examined TNFα and IL‐1β expression in peripheral blood and brain tissue. However, we found no evidence of pro‐inflammatory activity compared to 5×FAD mice (Figure 4B,C). The components of the NLRP3 inflammasome, ASC, and NLRP3 were also not significantly different between DNT‐treated mice and controls (Figure 4C).

To investigate the effect of DNT cell treatment on AD mice or their immune response in‐depth, we performed RNA sequencing on brain cells from the three groups of mice. DNT cell treatment caused significant alteration of the gene expression profile in comparison to the control 5×FAD mice (Figure 4D). Among the differentially expressed genes, *H2‐Aa* expression was downregulated in DNT‐treated mice compared to control 5×FAD mice, to a level similar to that of wild‐type mice (Figure 4E). *H2‐Aa* is a pro‐inflammatory factor that primarily responds to IFN‐γ produced by activated T cells and NK cells, and promotes antigen presentation and T‐cell activation [40, 41]. The decreased expression of this gene suggests that DNT cell treatment prevented responses to T cells or NK‐T cells, and attenuated the inflammatory response in DNT‐treated mice. Additionally, the marked rise in *Skap2* (Figure 4E) was able to regulate the activation of the immune system appropriately [42, 43].

In conclusion, DNT cell treatment exerts an immunomodulatory function in AD mice that fine‐tunes the extent of inflammation favoring neuroprotection. Our findings suggest that DNT cell treatment does not exacerbate inflammation and that its beneficial effects are mediated by the downregulation of *H2‐Aa* and upregulation of *Skap2*, which fine‐tune the inflammatory response.

### 3.5 DNT Cell Treatment Improves the Synaptic Plasticity and Increases the Complexity of Neurons

**FIGURE 5:**
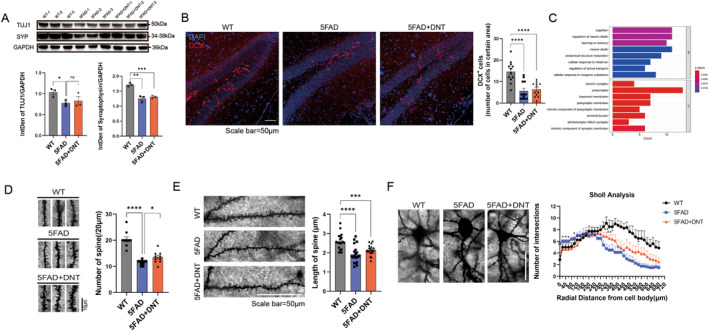
(A) Proteins of Tuj1 and synaptophysin expression levels of the three groups of mice in brain tissues by western blot. (B) Representative confocal images of *DCX* (Doublecortin) immunostaining in the hippocampus of the three groups of mice (left) and the number of DCX^+^ cells in the certain area were calculated (right). Scale bar = 50 μm; *n* = 12 views from 3 mice per group; mean ± SEM, ****p < 0.0001; unpaired Student's *t*‐test. (C) The histogram shows the up‐regulation gene of GO enrichment analysis of bulk RNA‐seq of the DNT‐treated mice versus the AD mice in brain tissue (Figure 4E). (D) Golgi staining of dendritic spines in neurons of the three groups mice cortex (left) and spine density was calculated (right) of 20 μm per view. Scale bar = 10 μm; *n* = 10 views from 2 mice per group; mean ± SEM, *p < 0.05; ****p < 0.0001; unpaired Student's *t*‐test. (E) The representative view of 100 μm neuron dendrites groups mice cortex(left), the histogram shows the statistics information of the length of spines on the dendrites(right). Scale bar = 50 μm; *n* = 20 values from 2 mice per group; mean ± SEM, ***p < 0.001; ****p < 0.0001; unpaired Student's *t*‐test. (F) The representative view of neuronal cell bodies and dendrites in the three groups of mice cortex (left), Sholl analysis of reconstructed neurons by simple neurite tracer (SNT) shows the complexity of neurons. Scale bar = 50 μm; *n* = 8 neurons from 2 mice per group; mean ± SEM, *p < 0.05; **p < 0.01; unpaired Student's *t*‐test.

To investigate the mechanism by which DNT infusion improves cognition in AD mice, we examined its effect on the neuronal state. As 5xFAD mice age, the sequential occurrence of synaptic dysfunction and neuronal loss deteriorates the basal synaptic transmission and LTP [44]. Therefore, activation of neurogenesis that replenishes the lost neurons may improve memory impairment. However, we found no significant differences in the expression of neural progenitor marker doublecortin or the neuronal‐related protein Tuj1 between the 5xFAD and DNT‐treated groups, indicating that cognitive improvement in DNT‐treated mice may not be induced by direct neuronal regeneration (Figure 5B,C).

We then investigated how DNT cell treatment alters synaptic growth and axonal lengthening by examining the expression of related genes such as *Cbln4*, *Ntn5*, *Prph*, and *Sema3b* [44, 45, 46] (Figure 4E). DNT cell treatment upregulated genes related to synaptic growth and axonal lengthening while downregulating genes that inhibit axonal extension, suggesting that DNT cell treatment regulates genes that act at synapses and presynaptic membranes to facilitate cognitive recovery, improve learning and memory, and regulate neural death (Figure 5A).

To assess the effect of DNT cell treatment on synaptic plasticity, we examined the expression and distribution of the presynaptic membrane synaptophysin protein and performed Golgi staining to assess the number and morphology of dendritic spines, which can affect synaptic plasticity and learning and memory ability [47, 48, 49]. We found no significant differences in the expression or distribution of the presynaptic membrane synaptophysin protein between the DNT‐treated and control groups (Figure 5B). However, DNT cell treatment significantly increased the number of dendritic spines without affecting their length, potentially favoring synaptic plasticity (Figure 5D,E).

Sholl analysis [50] revealed that DNT‐treated mice exhibited significantly more branches at the distal end of their neurites after treatment, to a level close to that of wild‐type mice (Figure 5F). This indicates that DNT cell treatment can potentially recover the dendrite field. However, in the control 5xFAD mice, more branches were concentrated in the proximal end of the cell body. Our findings suggest that DNT treatment increases dendritic spine density and neural complexity in mice rather than improving nerve regeneration ability, which accounts for the improvement of cognitive ability.

### 3.6 DNT Cell Treatment Decreases the Density of Amyloid Beta Plaques Deposition in the Cortex and Hippocampus of 5×FAD Mice

**FIGURE 6:**
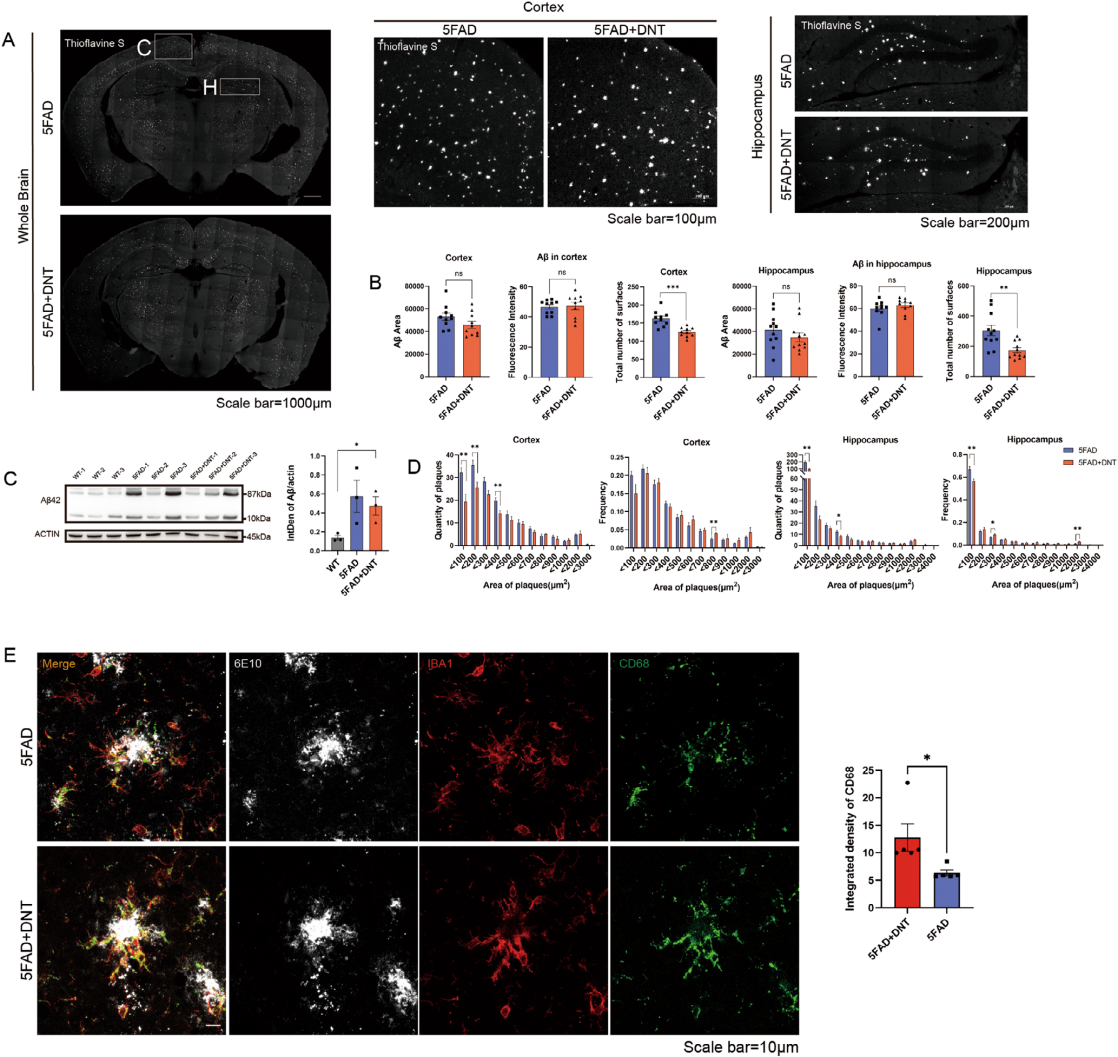
(A, B) Representative images of thioflavin‐S staining and quantification of the areas and fluorescence intensity and total numbers of Aβ plaques in the cortex and hippocampus; 5FAD, 5×FAD mice IV saline administration; 5FAD+DNT, 5×FAD+DNT, 5×FAD mice IV DNT administration. *n* = 15 slices from 3 mice per group; data are present as means ± SEM; ns, nonsignificant; *p < 0.05; **p < 0.01; ***p < 0.001; unpaired Student's *t*‐test. (C) Representative Western blots (left) and relative quantification (right) of Aβ‐42 expression levels in cortical tissues from the three groups. There was no significant reduction in Aβ‐42 deposition in 5×FAD and DNT‐treated mice. *n* = 3 mice in each group; mean ± SEM, ns, nonsignificant; *p < 0.05; one‐way ANOVA with Tukey's correction. (D) Based on the results (A and B), the quantities and frequency of plaques in the cortex and hippocampus of the two groups are shown in the histograms, every bar of the histogram represents the average values (upper) and average frequency [quantities of occurrence in the section/total number] of Aβ between the sections. Data are presented as means ± SEM; *p < 0.05; **p < 0.01; unpaired Student's *t*‐test. (E) Representative confocal images of Aβ1‐16 (6E10), lysosome (CD68), and microglia (IBA1) immunostaining in the cortex of the two groups of mice. *n* = 3 mice per group. Scale bar = 10 μm. Representative Western blots (right) and relative quantification of CD68 expression levels in cortical tissues from the three groups.

As the deposition of Aβ plaques is a characteristic pathological feature of AD that can contribute to cognitive decline [2, 51], we investigated how treatment with DNT may affect the stochastic of Aβ plaque, alleviate cognitive deficits, and enhance inter‐neuronal communication. Upon DNT cell treatment, we found a significant decrease in the number of plaques in both the cortex and hippocampus, although the fluorescence intensity of thioflavin S and 6E10 remained unchanged (Figures 6A and S2A). The levels of Aβ‐42 and expression of Aβ1‐16 in the brain were not affected by DNT cell treatment (Figures 6C and S2B). These findings suggest that DNT cells may regulate the number of plaques rather than their overall size. Consistent with this hypothesis, we observed a significant decrease in plaque counts in multiple brain regions of different sizes, such as in the range of 300–400, 100–200, and < 100 μm^2^ area of the cortex, as well as in the range of 300–400 and < 100 μm^2^ area of the hippocampus in the DNT treatment group compared to the 5×FAD mice (Figure 6D). These results may be associated with the downregulation of *Klk6* expression (Figure 4E), which encodes a protease that cleaves the amyloid precursor protein and alpha‐synuclein, potentially implicated in Alzheimer's and Parkinson's disease, respectively [52, 53]. This downregulation may have reduced the production of small plaques. Overall, DNT cell treatment significantly decreased the number of Aβ plaques in the cortex and hippocampus of AD mice, rendering them less harmful.

In AD, microglia are activated by the accumulation of Aβ plaques, leading to inflammation, apoptosis of surrounding cells, and damage to cholinergic neurons [54, 55]. On the other hand, when diffuse plaques become dense, the damage to the brain is reduced [56]. The increased stiffness of aggregated plaques enhances the tropism of microglia, promoting the clearance of Aβ in the brain and reducing its accumulation. Additionally, Aβ accumulation is associated with Aβ and Tau pathology seed propagation, and denser plaques may mitigate Aβ and Tau seed propagation between microglia and neurons [56, 57, 58]. We observed that DNT treatment led to denser plaques in the cortex, which may better recruit activated microglia and facilitate their phagocytosis through lysosomes (Figure 6E) but expression of CD68 in the whole brain had little change (Figure S2C). Furthermore, we also detected the clearance of 293T cell lines expressing tau by co‐culture with DNT in vitro (Figure S2D), a significant decrease in the AT8 was observed, indicating that DNT also has the ability to promote the clearance of tau. In summary, DNT cell treatment enhances the denseness of Aβ plaques in the cortex and hippocampus reduces their diffusion, and mitigates damage to the organism.

## 4 Discussion

In this study, we employed in vitro transformed DNT cells to treat 5×FAD mice through tail vein injection, and observed improvements in cognition and memory through increased synapse number and neural complexity, as well as an altered neuroprotection‐related state of microglia.

Alzheimer's disease is a complex neuroinflammatory disease with progressive pathological manifestations. Cell therapy using DNT cells, which inhibit inflammation, presents a promising approach for treating such diseases. DNT cells have several advantages over other cells used in cell therapy, such as MSC and NSC cells. They can be amplified in large quantities in vitro, and there is a lower risk of tumor formation, as they are not stem cells. Additionally, autologous transplantation of adult DNT cells is possible without the need to consider HVGR or GVHR, provided that the technology is up to date.

However, DNT cells may themselves have pro‐inflammatory effects, particularly after in vitro transformation and amplification, which raises concerns regarding their practical application in therapy. Previous studies have reported that DNT cells in patients or mouse models of stroke or AD exhibit significantly elevated levels of inflammatory factors [16, 39], indicating that they possess pro‐inflammatory properties in these diseases. In accordance with this, some diseases have shown to be less responsive to treatment with DNT cells [11, 37, 59]. Nonetheless, the infusion of in vitro CD4^+^ T cell‐derived DNT cells into AD mice resulted in significant cognitive improvements, which could be attributed to a subpopulation of DNT cells that removed the NK1.1 positive T cells and TCR γδ^+^ T cells. Naïve DNT cells that have not undergone proliferation have a reduced pro‐inflammatory effect and produce fewer inflammatory factors in vitro [60], making them a more practical option for cell therapy in the future. Obtaining sufficient quantities of the effective subset of naïve DNT cells from blood alone appears to be a promising avenue for future exploration.

CD4^+^ T cell‐derived DNT cells may exert their therapeutic effects through various avenues, as seen in existing cases where DNT cells were effective. For example, in the treatment of nonalcoholic steatohepatitis, self‐protection is mainly through IL‐10 secretion by M2‐like macrophages, while in stroke, it is mainly through elevated CCR5 expression and chemotactic DNT infiltration into the lesion site to exert inflammatory protection [12, 38]. In our study, DNT infusion likely improved the neuroprotective function of microglia, but the underlying mechanisms remain to be elucidated.

Our study also revealed some interesting findings that require further investigation, such as the shift of microglia to a neural‐associated state [61, 62]. If such a transition state of microglia exists, it is intriguing whether DNT exerts their function through direct infiltration into the brain and interaction with microglia, or through releasing factors and generating systemic effects. It is also interesting that DNT infusion upregulated some genes related to lifespan regulation, such as *Hsf1*. This suggests that DNT injection may potentially rejuvenate and prolong the lifespan of mice [63].

### 4.1 Research Limitations

DNT cell therapy has shown potential as a therapeutic option for Alzheimer's disease (AD). However, there are limitations to consider, including the need for further research to determine the safety and efficacy of using CD4^+^ T cell‐derived DNT cells in human patients with AD. The mechanism by which DNT cells exert their therapeutic effects is not fully understood, and sourcing and preparing DNT cells can be challenging and expensive, limiting their widespread use. Additionally, DNT cell therapy may not be effective for all patients with AD, as the disease is complex and multifactorial with varying underlying causes and mechanisms of disease progression.

## Author Contributions

Y.X., J.L., Z.H., and H.W. collection and assembly of data, manuscript writing; K.L., X.C., Z.F., D.L., C.L. collection and assembly of data; Y.P. provided cell sources; Yu.Z. DNT transplantation and collection of data; Ya.Z. and B.H. conception and design, data analysis and interpretation, final approval of the manuscript.

## Conflicts of Interest

The authors declare no conflicts of interest.

## Supporting information


**FIGURE S1.** (A) Results of the open‐field test for the three groups of female mice. (B) Unbiased identification of cell‐type heterogeneity in the DNT‐treated mice cortex (left), and microglial cell populations are marked in red. UMAP harmony showing the microglia cluster of the three groups of mice (WT, wild‐type mice intravenous (IV) saline administration; AD, 5×FAD mice IV saline administration; DNT, 5×FAD mice IV DNT administration) (right). *n* = 3 mice per group for single‐cell sequencing, analyzed by Seurat (R package, 4.1.0). (C) Volcano plot depicting changes in gene expression in microglia owing to DNT treatment compared to 5×FAD mice (upper). The *x*‐axis corresponds to log_2_ (fold change in gene expression), and the *y*‐axis indicates the adjusted *p*‐value. The genes colored red have the symbol as follows: log_2_ fold change > 1 or < − 1 and adjusted *p*‐value < 0.05, and genes colored blue meet the standard of the log2 fold change < 1 or > − 1 and adjusted *p*‐value < 0.05. Bar graph showing enriched ontology terms for significantly upregulated genes (log2 fold change > 1 and *p*‐value < 0.05) in DNT‐treated mice relative to 5×FAD mice microglia (lower). (D) UMAP plots of the microglia in (A, right). UMAP plots are colored by the expression of key marker genes, *Apoe* (upper) and *Trem2* (lower). (E) UMAP plots of the microglia in (A, right). Distribution of the three groups of mice hippocampal microglia into nine distinct clusters (left). Quantification of the different clusters of microglia (right) of the three groups. (F) Markers label the nine clusters shown, the color bar represents the average expression of genes, and the circle size represents the percentage of gene expression. The numbers 0–8 are consistent in the three graphs, representing the nine microglia clusters.

**FIGURE S2.** (A) Representative images of 6E10 staining in the cortex and hippocampus; 5FAD, 5×FAD mice IV saline administration; 5FAD+DNT, 5×FAD+DNT, 5×FAD mice IV DNT administration. *n* = 8 slices from 3 mice per group; data are presented means ± SEM; ns, nonsignificant; *p < 0.05; **p < 0.01; ***p < 0.001; unpaired Student’s *t* test. (B) Representative Western blots (left) and relative quantification (right) of 6E10 expression levels in cortical tissues from the three groups. There was no significant reduction in 6E10 deposition in 5×FAD and DNT‐treated mice. *n* = 3 mice in each group; mean ± SEM, ns, nonsignificant; *p < 0.05; one‐way ANOVA with Tukey’s correction. (C) Representative Western blots (upper) and relative quantification (lower) of 6E10 expression levels in the whole brain from the three groups. (D) Representative images of AT8 staining in the 293T‐tau‐GFP cell lines co‐culturing with nothing or DNT in vitro and the proportion of AT8^+^ cells statistical graph.

## Data Availability

The data supporting this study's findings are available from the corresponding author upon reasonable request.

## References

[1] J. M. Long and D. M. Holtzman , “Alzheimer Disease: An Update on Pathobiology and Treatment Strategies,” Cell 179, no. 2 (2019): 312–339, 10.1016/j.cell.2019.09.001. 31564456 PMC6778042

[2] B. Zott , M. M. Simon , W. Hong , et al., “A Vicious Cycle of β Amyloid–Dependent Neuronal Hyperactivation,” Science 365, no. 6453 (2019): 559–565, 10.1126/science.aay0198. 31395777 PMC6690382

[3] R. Sankowski , C. Böttcher , T. Masuda , et al., “Mapping Microglia States in the Human Brain Through the Integration of High‐Dimensional Techniques,” Nature Neuroscience 22, no. 12 (2019): 2098–2110, 10.1038/s41593-019-0532-y. 31740814

[4] Y. Chen and M. Colonna , “Two‐Faced Behavior of Microglia in Alzheimer's Disease,” Nature Neuroscience 25, no. 1 (2022): 3–4, 10.1038/s41593-021-00963-w. 34815557

[5] F. Leng and P. Edison , “Neuroinflammation and Microglial Activation in Alzheimer Disease: Where Do We Go From Here?,” Nature Reviews Neurology 17, no. 3 (2021): 157–172, 10.1038/s41582-020-00435-y. 33318676

[6] L. Jia , M. Quan , Y. Fu , et al., “Dementia in China: Epidemiology, Clinical Management, and Research Advances,” Lancet Neurology 19, no. 1 (2020): 81–92, 10.1016/S1474-4422(19)30290-X. 31494009

[7] R. Ren , J. Qi , S. Lin , et al., “The China Alzheimer Report 2022,” General Psychiatry 35, no. 1 (2022): e100751, 10.1136/gpsych-2022-100751. 35372787 PMC8919463

[8] J. Liu , Z. Hou , J. Wu , et al., “Infusion of hESC Derived Immunity‐and‐Matrix Regulatory Cells Improves Cognitive Ability in Early‐Stage AD Mice,” Cell Proliferation 54, no. 8 (2021): e13085, 10.1111/cpr.13085. 34232542 PMC8349653

[9] A. S. Cone , X. Yuan , L. Sun , et al., “Mesenchymal Stem Cell‐Derived Extracellular Vesicles Ameliorate Alzheimer's Disease‐Like Phenotypes in a Preclinical Mouse Model,” Theranostics 11, no. 17 (2021): 8129–8142, 10.7150/thno.62069. 34373732 PMC8344012

[10] G. Santamaria , E. Brandi , P. L. Vitola , et al., “Intranasal Delivery of Mesenchymal Stem Cell Secretome Repairs the Brain of Alzheimer's Mice,” Cell Death and Differentiation 28, no. 1 (2021): 203–218, 10.1038/s41418-020-0592-2. 32704089 PMC7852675

[11] C. Han , Y. Sheng , J. Wang , et al., “Double‐Negative T Cells Mediate M1 Polarization of Microglial Cells via TNF‐α‐NLRP3 to Aggravate Neuroinflammation and Cognitive Impairment in Alzheimer's Disease Mice,” Journal of Cellular Physiology 237 (2022): 3860–3871, 10.1002/jcp.30839. 35866513

[12] D. Tian , Y. Pan , Y. Zhao , et al., “TCRαβ^+^ NK1.1^−^ CD4^−^ CD8^−^ Double‐Negative T Cells Inhibit Central and Peripheral Inflammation and Ameliorate Ischemic Stroke in Mice,” Theranostics 13, no. 3 (2023): 896–909, 10.7150/thno.80307. 36793857 PMC9925325

[13] D. Zhang , W. Yang , N. Degauque , Y. Tian , A. Mikita , and X. X. Zheng , “New Differentiation Pathway for Double‐Negative Regulatory T Cells That Regulates the Magnitude of Immune Responses,” Blood 109, no. 9 (2007): 4071–4079, 10.1182/blood-2006-10-050625. 17197428 PMC1874581

[14] D. Zhang , W. Zhang , T. W. Ng , et al., “Adoptive Cell Therapy Using Antigen‐Specific CD4−CD8− T Regulatory Cells to Prevent Autoimmune Diabetes and Promote Islet Allograft Survival in NOD Mice,” Diabetologia 54, no. 8 (2011): 2082–2092, 10.1007/s00125-011-2179-4. 21594554

[15] M. Antunes and G. Biala , “The Novel Object Recognition Memory: Neurobiology, Test Procedure, and Its Modifications,” Cognitive Processing 13, no. 2 (2012): 93–110, 10.1007/s10339-011-0430-z. 22160349 PMC3332351

[16] M. Nava Catorce , G. Acero , and G. Gevorkian , “Age‐ and Sex‐Dependent Alterations in the Peripheral Immune System in the 3xTg‐AD Mouse Model of Alzheimer's Disease: Increased Proportion of CD3+CD4‐CD8‐ Double‐Negative T Cells in the Blood,” Journal of Neuroimmunology 360 (2021): 577720, 10.1016/j.jneuroim.2021.577720. 34543880

[17] S. Prokop , K. R. Miller , and F. L. Heppner , “Microglia Actions in Alzheimer's Disease,” Acta Neuropathologica 126, no. 4 (2013): 461–477, 10.1007/s00401-013-1182-x. 24224195

[18] A. Mikdache , L. Fontenas , S. Albadri , et al., “Elmo1 Function, Linked to Rac1 Activity, Regulates Peripheral Neuronal Numbers and Myelination in Zebrafish,” Cellular and Molecular Life Sciences 77, no. 1 (2020): 161–177, 10.1007/s00018-019-03167-5. 31161284 PMC11104998

[19] M. Blazejczyk , M. Macias , M. Korostynski , et al., “Kainic Acid Induces mTORC1‐Dependent Expression of Elmo1 in Hippocampal Neurons,” Molecular Neurobiology 54, no. 4 (2017): 2562–2578, 10.1007/s12035-016-9821-6. 26993296 PMC5390005

[20] S. Krasemann , C. Madore , R. Cialic , et al., “The TREM2‐APOE Pathway Drives the Transcriptional Phenotype of Dysfunctional Microglia in Neurodegenerative Diseases,” Immunity 47, no. 3 (2017): 566–581.e9, 10.1016/j.immuni.2017.08.008. 28930663 PMC5719893

[21] A. T. Nguyen , K. Wang , G. Hu , et al., “APOE and TREM2 Regulate Amyloid‐Responsive Microglia in Alzheimer's Disease,” Acta Neuropathologica 140, no. 4 (2020): 477–493, 10.1007/s00401-020-02200-3. 32840654 PMC7520051

[22] D. Sepulveda‐Falla , J. S. Sanchez , M. C. Almeida , et al., “Distinct Tau Neuropathology and Cellular Profiles of an APOE3 Christchurch Homozygote Protected Against Autosomal Dominant Alzheimer's Dementia,” Acta Neuropathologica 144, no. 3 (2022): 589–601, 10.1007/s00401-022-02467-8. 35838824 PMC9381462

[23] K. Maeda , S. Matsuhashi , K. Tabuchi , et al., “Brain Specific Human Genes, NELL1 and NELL2, Are Predominantly Expressed in Neuroblastoma and Other Embryonal Neuroepithelial Tumors,” Neurologia Medico‐Chirurgica 41, no. 12 (2001): 582–589, 10.2176/nmc.41.582. 11803583

[24] K. Yamamoto , V. Gandin , M. Sasaki , et al., “Largen: A Molecular Regulator of Mammalian Cell Size Control,” Molecular Cell 53, no. 6 (2014): 904–915, 10.1016/j.molcel.2014.02.028. 24656129

[25] F. Gueniot , S. Rubin , P. Bougaran , et al., “Targeting Pdzrn3 Maintains Adult Blood‐Brain Barrier and Central Nervous System Homeostasis,” Journal of Cerebral Blood Flow and Metabolism 42, no. 4 (2022): 613–629, 10.1177/0271678X211048981. 34644209 PMC9051145

[26] R. Suzuki , A. Fujikawa , Y. Komatsu , K. Kuboyama , N. Tanga , and M. Noda , “Enhanced Extinction of Aversive Memories in Mice Lacking SPARC‐Related Protein Containing Immunoglobulin Domains 1 (SPIG1/FSTL4),” Neurobiology of Learning and Memory 152 (2018): 61–70, 10.1016/j.nlm.2018.05.010. 29783061

[27] Y. Liu , G. J. Zou , B. X. Tu , et al., “Corticosterone Induced the Increase of proBDNF in Primary Hippocampal Neurons via Endoplasmic Reticulum Stress,” Neurotoxicity Research 38, no. 2 (2020): 370–384, 10.1007/s12640-020-00201-4. 32378057

[28] A. Deczkowska , H. Keren‐Shaul , A. Weiner , M. Colonna , M. Schwartz , and I. Amit , “Disease‐Associated Microglia: A Universal Immune Sensor of Neurodegeneration,” Cell 173, no. 5 (2018): 1073–1081, 10.1016/j.cell.2018.05.003. 29775591

[29] M. Kenigsbuch , P. Bost , S. Halevi , et al., “A Shared Disease‐Associated Oligodendrocyte Signature Among Multiple CNS Pathologies,” Nature Neuroscience 25, no. 7 (2022): 876–886, 10.1038/s41593-022-01104-7. 35760863 PMC9724210

[30] J. S. Sadick , M. R. O'Dea , P. Hasel , T. Dykstra , A. Faustin , and S. A. Liddelow , “Astrocytes and Oligodendrocytes Undergo Subtype‐Specific Transcriptional Changes in Alzheimer's Disease,” Neuron 110 (2022): 1788–1805.e10, 10.1016/j.neuron.2022.03.008. 35381189 PMC9167747

[31] S. Pandey , K. Shen , S. H. Lee , et al., “Disease‐Associated Oligodendrocyte Responses Across Neurodegenerative Diseases,” Cell Reports 40, no. 8 (2022): 111189, 10.1016/j.celrep.2022.111189. 36001972

[32] J. M. Taylor , Y. J. C. Song , Y. Huang , et al., “Parkin Co‐Regulated Gene (PACRG) is Regulated by the Ubiquitin–Proteasomal System and Is Present in the Pathological Features of Parkinsonian Diseases,” Neurobiology of Disease 27, no. 2 (2007): 238–247, 10.1016/j.nbd.2007.04.014. 17590346

[33] J. M. Nerbonne , B. R. Gerber , A. Norris , and A. Burkhalter , “Electrical Remodelling Maintains Firing Properties in Cortical Pyramidal Neurons Lacking *KCND2*‐Encoded A‐Type K ^+^ Currents,” Journal of Physiology 586, no. 6 (2008): 1565–1579, 10.1113/jphysiol.2007.146597. 18187474 PMC2375705

[34] D. Subramaniam , S. Ramalingam , D. C. Linehan , et al., “RNA Binding Protein CUGBP2/CELF2 Mediates Curcumin‐Induced Mitotic Catastrophe of Pancreatic Cancer Cells,” PLoS ONE 6, no. 2 (2011): e16958, 10.1371/journal.pone.0016958. 21347286 PMC3037932

[35] Z. C. Wu , J. T. Yu , N. D. Wang , et al., “Lack of Association Between PCDH11X Genetic Variation and Late‐Onset Alzheimer's Disease in a Han Chinese Population,” Brain Research 1357 (2010): 152–156, 10.1016/j.brainres.2010.08.008. 20707987

[36] H. Yuan , Q. Wang , Y. Liu , et al., “A Rare Exonic *NRXN3* Deletion Segregating With Neurodevelopmental and Neuropsychiatric Conditions in a Three‐Generation Chinese Family,” American Journal of Medical Genetics 177, no. 6 (2018): 589–595, 10.1002/ajmg.b.32673. 30076746 PMC6445570

[37] H. Meng , H. Zhao , X. Cao , et al., “Double‐Negative T Cells Remarkably Promote Neuroinflammation After Ischemic Stroke,” Proceedings of the National Academy of Sciences of the United States of America 116, no. 12 (2019): 5558–5563, 10.1073/pnas.1814394116. 30819895 PMC6431175

[38] G. Sun , X. Zhao , M. Li , et al., “CD4 Derived Double Negative T Cells Prevent the Development and Progression of Nonalcoholic Steatohepatitis,” Nature Communications 12, no. 1 (2021): 650, 10.1038/s41467-021-20941-x. PMC7844244 33510172

[39] H. Wood , “A Role for Double‐Negative T Cells in Post‐Stroke Neuroinflammation,” Nature Reviews Neurology 15, no. 5 (2019): 246–247, 10.1038/s41582-019-0171-7. 30886394

[40] P. Yang , Q. Wu , L. Sun , et al., “Adaptive Immune Response Signaling Is Suppressed in Ly6Chigh Monocyte but Upregulated in Monocyte Subsets of ApoE −/− Mice — Functional Implication in Atherosclerosis,” Frontiers in Immunology 12 (2021): 809208, 10.3389/fimmu.2021.809208. 34987524 PMC8721109

[41] Y. Zhao , J. Xiong , H. X. Chen , et al., “A Spontaneous H2‐Aa Point Mutation Impairs MHC II Synthesis and CD4+ T‐Cell Development in Mice,” Frontiers in Immunology 13 (2022): 810824, 10.3389/fimmu.2022.810824. 35309308 PMC8931304

[42] N. Rutsch , C. E. Chamberlain , W. Dixon , et al., “Diabetes With Multiple Autoimmune and Inflammatory Conditions Linked to an Activating SKAP2 Mutation,” Diabetes Care 44, no. 8 (2021): 1816–1825, 10.2337/dc20-2317. 34172489 PMC8385470

[43] M. Tanaka , S. Shimamura , S. Kuriyama , D. Maeda , A. Goto , and N. Aiba , “SKAP2 Promotes Podosome Formation to Facilitate Tumor‐Associated Macrophage Infiltration and Metastatic Progression,” Cancer Research 76, no. 2 (2016): 358–369, 10.1158/0008-5472.CAN-15-1879. 26577701

[44] G. Esquerda‐Canals , L. Montoliu‐Gaya , J. Güell‐Bosch , and S. Villegas , “Mouse Models of Alzheimer's Disease,” Journal of Alzheimer's Disease 57, no. 4 (2017): 1171–1183, 10.3233/JAD-170045. 28304309

[45] Y. Du , Y. Shi , X. Wang , et al., “Hippocampal Semaphorin 3B Improves Depression‐Like Behaviours in Mice by Upregulating Synaptic Plasticity and Inhibiting Neuronal Apoptosis,” Journal of Neurochemistry 163, no. 2 (2022): 133–148, 10.1111/jnc.15680. 35892177

[46] K. Liakath‐Ali , J. S. Polepalli , S. J. Lee , J. F. Cloutier , and T. C. Südhof , “Transsynaptic Cerebellin 4–Neogenin 1 Signaling Mediates LTP in the Mouse Dentate Gyrus,” Proceedings of the National Academy of Sciences of the United States of America 119, no. 20 (2022): e2123421119, 10.1073/pnas.2123421119. 35544694 PMC9171784

[47] S. Ikegaya , Y. Iga , S. Mikawa , et al., “Decreased Proliferation in the Neurogenic Niche, Disorganized Neuroblast Migration, and Increased Oligodendrogenesis in Adult Netrin‐5‐Deficient Mice,” Frontiers in Neuroscience 14 (2020): 570974, 10.3389/fnins.2020.570974. 33324143 PMC7726356

[48] D. Sabbatini , F. Raggi , S. Ruggero , et al., “Evaluation of Peripherin in Biofluids of Patients With Motor Neuron Diseases,” Annals of Clinical Translational Neurology 8, no. 8 (2021): 1750–1754, 10.1002/acn3.51419. 34264016 PMC8351396

[49] T. C. Südhof , “The Cell Biology of Synapse Formation,” Journal of Cell Biology 220, no. 7 (2021): e202103052, 10.1083/jcb.202103052. 34086051 PMC8186004

[50] K. E. Binley , W. S. Ng , J. R. Tribble , B. Song , and J. E. Morgan , “Sholl Analysis: A Quantitative Comparison of Semi‐Automated Methods,” Journal of Neuroscience Methods 225 (2014): 65–70, 10.1016/j.jneumeth.2014.01.017. 24485871

[51] H. Oakley , S. L. Cole , S. Logan , et al., “Intraneuronal β‐Amyloid Aggregates, Neurodegeneration, and Neuron Loss in Transgenic Mice With Five Familial Alzheimer's Disease Mutations: Potential Factors in Amyloid Plaque Formation,” Journal of Neuroscience 26, no. 40 (2006): 10129–10140, 10.1523/JNEUROSCI.1202-06.2006. 17021169 PMC6674618

[52] O. Goldhardt , I. Warnhoff , I. Yakushev , et al., “Kallikrein‐Related Peptidases 6 and 10 Are Elevated in Cerebrospinal Fluid of Patients With Alzheimer's Disease and Associated With CSF‐TAU and FDG‐PET,” Translational Neurodegeneration 8, no. 1 (2019): 25, 10.1186/s40035-019-0168-6. 31467673 PMC6712703

[53] K. Patra , A. Soosaipillai , S. B. Sando , et al., “Assessment of Kallikrein 6 as a Cross‐Sectional and Longitudinal Biomarker for Alzheimer's Disease,” Alzheimer's Research & Therapy 10, no. 1 (2018): 9, 10.1186/s13195-018-0336-4. PMC5789599 29378650

[54] Y. Huang , K. E. Happonen , P. G. Burrola , et al., “Microglia Use TAM Receptors to Detect and Engulf Amyloid β Plaques,” Nature Immunology 22, no. 5 (2021): 586–594, 10.1038/s41590-021-00913-5. 33859405 PMC8102389

[55] F. A. Edwards , “A Unifying Hypothesis for Alzheimer's Disease: From Plaques to Neurodegeneration,” Trends in Neurosciences 42, no. 5 (2019): 310–322, 10.1016/j.tins.2019.03.003. 31006494

[56] P. d'Errico , S. Ziegler‐Waldkirch , V. Aires , et al., “Microglia Contribute to the Propagation of Aβ Into Unaffected Brain Tissue,” Nature Neuroscience 25, no. 1 (2022): 20–25, 10.1038/s41593-021-00951-0. 34811521 PMC8737330

[57] C. Wang , L. Fan , R. R. Khawaja , et al., “Microglial NF‐κB Drives Tau Spreading and Toxicity in a Mouse Model of Tauopathy,” Nature Communications 13, no. 1 (2022): 1969, 10.1038/s41467-022-29552-6. PMC9005658 35413950

[58] Y. Cai , J. Du , A. Li , et al., “Initial Levels of β‐Amyloid and Tau Deposition Have Distinct Effects on Longitudinal Tau Accumulation in Alzheimer's Disease,” Alzheimer's Research & Therapy 15 (2023): 30, 10.1186/s13195-023-01178-w. PMC9903587 36750884

[59] M. Bulati , S. Buffa , A. Martorana , et al., “Double Negative (IgG^+^ IgD^−^ CD27^−^) B Cells Are Increased in a Cohort of Moderate‐Severe Alzheimer's Disease Patients and Show a pro‐Inflammatory Trafficking Receptor Phenotype,” Journal of Alzheimer's Disease 44, no. 4 (2015): 1241–1251, 10.3233/jad-142412. 25408215

[60] L. Yang , Y. Zhu , D. Tian , et al., “Transcriptome Landscape of Double Negative T Cells by Single‐Cell RNA Sequencing,” Journal of Autoimmunity 121 (2021): 102653, 10.1016/j.jaut.2021.102653. 34022742

[61] T. Matsuda , T. Irie , S. Katsurabayashi , et al., “Pioneer Factor NeuroD1 Rearranges Transcriptional and Epigenetic Profiles to Execute Microglia‐Neuron Conversion,” Neuron 101, no. 3 (2019): 472–485.e7, 10.1016/j.neuron.2018.12.010. 30638745

[62] Y. Rao , S. Du , B. Yang , et al., “NeuroD1 Induces Microglial Apoptosis and Cannot Induce Microglia‐to‐Neuron Cross‐Lineage Reprogramming,” Neuron 109, no. 24 (2021): 4094–4108, 10.1016/j.neuron.2021.11.008. 34875233

[63] F. Chen , Y. Fan , P. Cao , et al., “Pan‐Cancer Analysis of the Prognostic and Immunological Role of HSF1: A Potential Target for Survival and Immunotherapy,” Oxidative Medicine and Cellular Longevity 2021 (2021): 5551036, 10.1155/2021/5551036. 34239690 PMC8238600

