# Supplementary figures and images for: CD4‐Derived Double‐Negative T Cells Ameliorate Alzheimer's Disease‐Like Phenotypes in the 5×FAD Mouse Model
Source: CNS Neurosci Ther. 2025 Jan 23;31(1):e70187. doi: 10.1111/cns.70187 (PMC11754964; doi:10.1111/cns.70187)

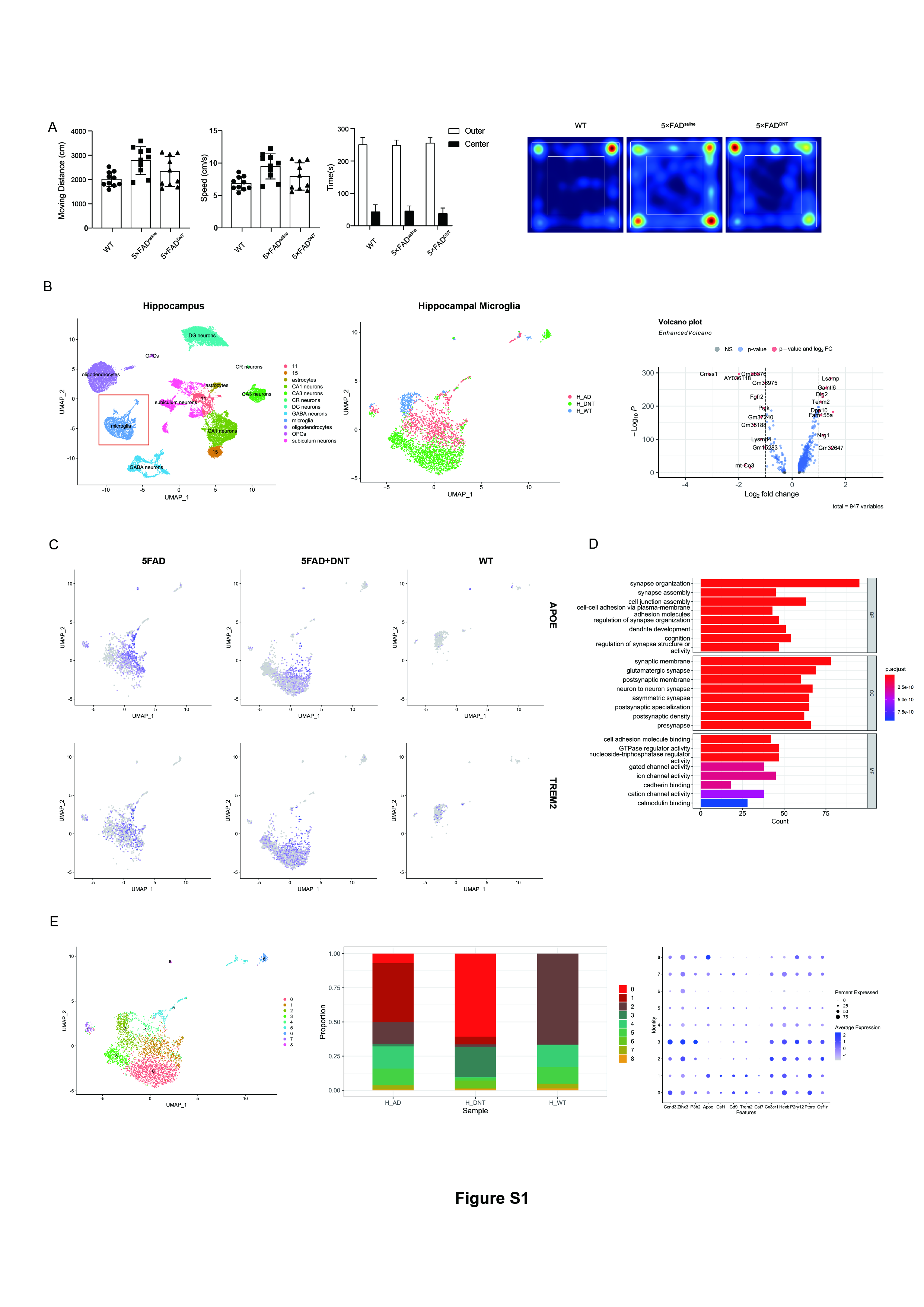

Supplement: Supplementary file 1 — FIGURE S1. (A) Results of the open‐field test for the three groups of female mice. (B) Unbiased identification of cell‐type heterogeneity in the DNT‐treated mice cortex (left), and microglial cell populations are marked in red. UMAP harmony showing the microglia cluster of the three groups of mice (WT, wild‐type mice intravenous (IV) saline administration; AD, 5×FAD mice IV saline administration; DNT, 5×FAD mice IV DNT administration) (right). n = 3 mice per group for single‐cell sequencing, analyzed by Seurat (R package, 4.1.0). (C) Volcano plot depicting changes in gene expression in microglia owing to DNT treatment compared to 5×FAD mice (upper). The x‐axis corresponds to log2 (fold change in gene expression), and the y‐axis indicates the adjusted p‐value. The genes colored red have the symbol as follows: log2 fold change > 1 or < − 1 and adjusted p‐value < 0.05, and genes colored blue meet the standard of the log2 fold change < 1 or > − 1 and adjusted p‐value < 0.05. Bar graph showing enriched ontology terms for significantly upregulated genes (log2 fold change > 1 and p‐value < 0.05) in DNT‐treated mice relative to 5×FAD mice microglia (lower). (D) UMAP plots of the microglia in (A, right). UMAP plots are colored by the expression of key marker genes, Apoe (upper) and Trem2 (lower). (E) UMAP plots of the microglia in (A, right). Distribution of the three groups of mice hippocampal microglia into nine distinct clusters (left). Quantification of the different clusters of microglia (right) of the three groups. (F) Markers label the nine clusters shown, the color bar represents the average expression of genes, and the circle size represents the percentage of gene expression. The numbers 0–8 are consistent in the three graphs, representing the nine microglia clusters. [file CNS-31-e70187-s002.tif]

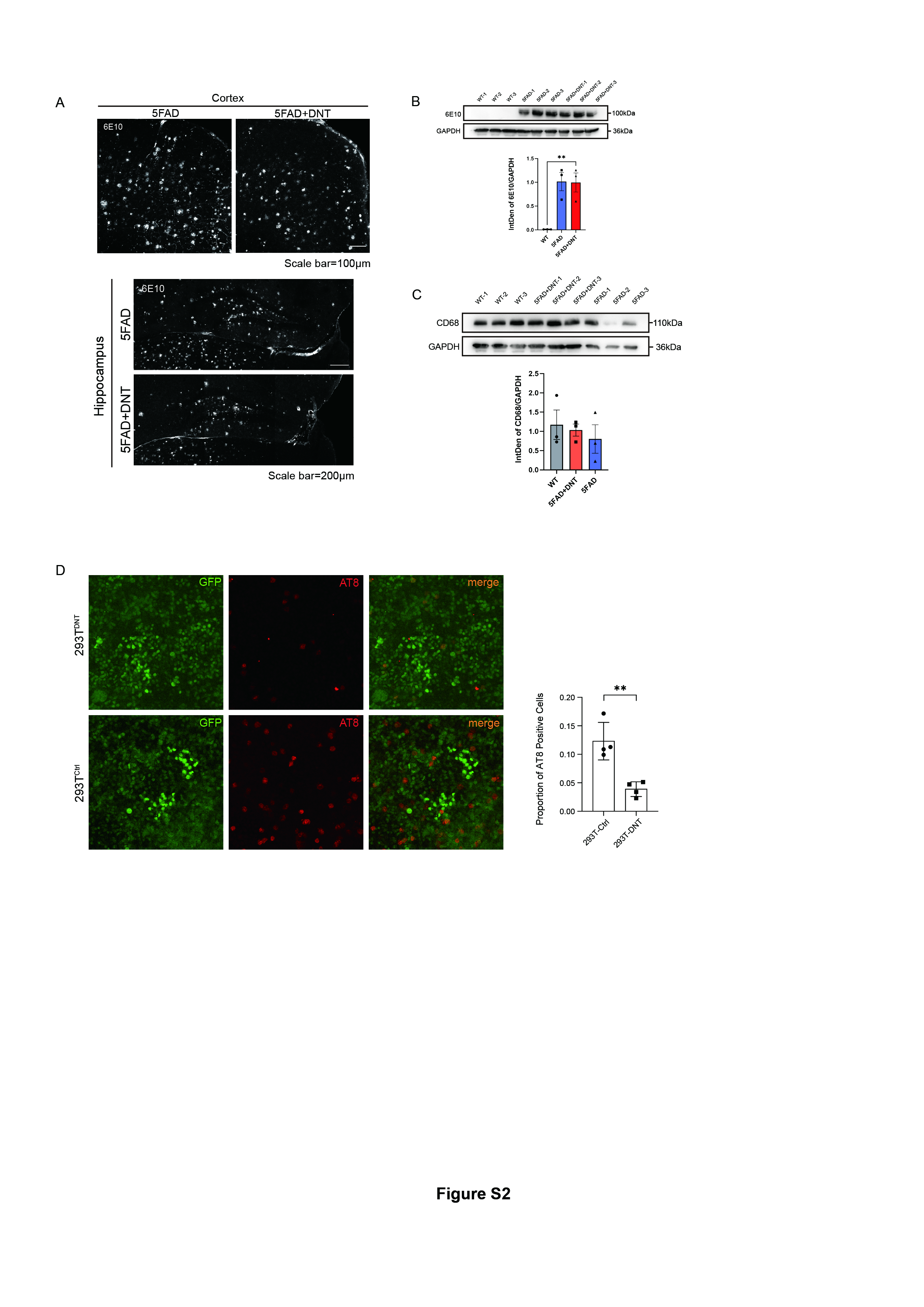

Supplement: Supplementary file 2 — FIGURE S2. (A) Representative images of 6E10 staining in the cortex and hippocampus; 5FAD, 5×FAD mice IV saline administration; 5FAD+DNT, 5×FAD+DNT, 5×FAD mice IV DNT administration. n = 8 slices from 3 mice per group; data are presented means ± SEM; ns, nonsignificant; *p < 0.05; **p < 0.01; ***p < 0.001; unpaired Student’s t test. (B) Representative Western blots (left) and relative quantification (right) of 6E10 expression levels in cortical tissues from the three groups. There was no significant reduction in 6E10 deposition in 5×FAD and DNT‐treated mice. n = 3 mice in each group; mean ± SEM, ns, nonsignificant; *p < 0.05; one‐way ANOVA with Tukey’s correction. (C) Representative Western blots (upper) and relative quantification (lower) of 6E10 expression levels in the whole brain from the three groups. (D) Representative images of AT8 staining in the 293T‐tau‐GFP cell lines co‐culturing with nothing or DNT in vitro and the proportion of AT8+ cells statistical graph. [file CNS-31-e70187-s001.tif]
